# Equivalent doses for anticancer agents used in pediatric oncology: A literature review and evaluation of a novel approach for conversion factors

**DOI:** 10.1002/cnr2.1811

**Published:** 2023-03-28

**Authors:** Meike Ressing, Cornelia Becker, Christian Müller, Seyed Hamidreza Mahmoudpour, Gabriele Calaminus, Thorsten Langer, Friederike Erdmann, Mathias Voigt, Melanie Kaiser, Peter Kaatsch, Maria Blettner, Claudia Spix

**Affiliations:** ^1^ German Childhood Cancer Registry (GCCR), Division of Childhood Cancer Epidemiology, Institute of Medical Biostatistics, Epidemiology and Informatics (IMBEI) University Medical Center of the Johannes Gutenberg‐University Mainz Mainz Germany; ^2^ Gert and Susanna Mayer Foundation Wuppertal Germany; ^3^ Department of Pediatric Hematology and Oncology University Hospital Bonn Bonn Germany; ^4^ Pediatric Oncology and Hematology University Hospital for Children and Adolescents Lübeck Germany; ^5^ Institute of Medical Biostatistics, Epidemiology and Informatics (IMBEI) University Medical Center of the Johannes Gutenberg‐University Mainz Mainz Germany

**Keywords:** chemotherapeutics, childhood cancer, conversion factors, doses, second tumor

## Abstract

**Background:**

Epidemiological research on late effects of therapy shows the necessity to aggregate chemotherapy agents to substance classes. This requires using conversion factors by substance classes.

**Aims:**

The aim of this study was to identify previously used conversion factors from the literature, to present a novel approach for additional factors, and to compare these approaches.

**Methods and Results:**

A literature review was performed, which identified two main principles of deriving conversion factors: effect‐equivalence and equimolar. Thirty‐five articles presenting effect equivalence‐based factors in the widest sense were found in the literature. Ten articles presented the equimolar approach which can be applied to almost all chemotherapy substances. Based on a comprehensive list of treatment protocols used in German pediatric oncology, we derived alternative conversion factors from typical doses. We compared the conversion factors using Pearson correlation coefficients and linear regression. At least two types of conversion factor were available for each of the 49 substances included. The equivalent effect‐based and the typical dose‐based factors were highly correlated with a regression coefficient close to 1. The equimolar factors are independent.

**Conclusions:**

For substances for which no conversion factor based on some type of effect equivalence has been published so far, a factor based on a typical doses‐approach may be used in epidemiological late effects research. Doses aggregated based on the equimolar approach may not be compatible with doses aggregated based on equivalent effects.

## 1 INTRODUCTION

Our working group is working on a case–control study on second neoplasms after childhood cancer (second tumors after tumor therapy (STATT)) using data from the German Childhood Cancer Registry (GCCR) and German clinical therapy trials in pediatric oncology, soon to be published). For this we obtained retrospective cumulative chemotherapy dose data for the former patients. It became clear that the number of different substances is too large for joint statistical analysis and some substances are applied rarely and therefore allow no statistical analysis. Other groups working on late effects of chemotherapy had been using the solution of grouping substances by pharmacologic principles,^1^, ^2^ usually using a conversion factor before aggregating cumulative doses in a substance group (e.g., References 3, 4, 5, 6, 7, 8). Clinical replacement rules require conversion factors, too.^9^, ^10^ Given the sometimes very different dose range of substances in a substance group, aggregating them without conversion is not indicated.

However, a comprehensive list of substances used in pediatric oncology and conversion factors for them turned out not to be available in the pertinent literature. Therefore, we initiated a very broad literature search aiming to collect factors having been used before in this field of late effects research, with a special focus on childhood cancer survivors. We are presenting the results of this search here.

In addition, we developed an algorithm to fill in conversion factors for which conversion factors cannot be found in our literature search. This approach is based on typical doses determined from a comprehensive list of treatment protocols of the German Society for Pediatric Oncology and Hematology (GPOH) from the years 1970 to 2018.^11^, ^12^ We are presenting these factors here, too. The final question was whether it is justified using conversion factors based on different principles in the same analyses; for this we compared the factors statistically.

## 2 METHODS

### 2.1 Inclusion and grouping of substances

We included all substances with reported conversion factors in the pertinent literature and which have been used in treatment protocols for pediatric oncology in Germany since the 1970s.^11^, ^12^ They were included if they are considered as antineoplastic agents (Group L01) according to the Anatomical Therapeutic Chemical (ATC) code,^2^ excluding immunotherapy and supportive substances. We also examined glucocorticoids which are used as antineoplastic agents in pediatric oncology although they are not listed as such according to the ATC (Group H02AB).^13^ Doses were given in or converted to the unit mg/m^2^ (except for asparaginase (L01XX), where International Units (IU)/m^2^ are generally used). The substances were classified into 12 substance groups according to the ATC.^2^ For each class, a reference substance was chosen. Based on the ATC, procarbazine and estramustine belong to the group ‘other antineoplastic agents’ (L01X). However, due to their mode of action, they are usually grouped with alkylating agents (L01A) in oncology literature.^3^, ^8^

### 2.2 Literature review

The literature review was performed as a scoping review according to the PRISMA‐ScR (Preferred Reporting Items for Systematic reviews and Meta‐Analyses extension for Scoping Reviews) checklist.^14^ The literature search was performed in Medline via PubMed on December 13th, 2022 and in Web of Science Core Collection on November 29th, 2022.

The search strategy with criteria for inclusion and exclusion was defined a priori (see Table 1). Given that we were mainly interested in applying this to research on secondary carcinogenicity in treated children, we used the following search terms: ‘childhood second cancer AND chemotherapy AND dose’ (Search 1). In order to include articles examining glucocorticoids as well, we performed an additional specific search using ‘cortisone AND equivalence dose’. The resulting queries are provided in detail in Supplementary Table 1.

**TABLE 1:** Inclusion and exclusion criteria for the literature review

Step	Inclusion criteria	Exclusion criteria
First step: Title and abstract	Published in 1985 or later	**–**
	Published in English or German	
	Examining the effect of different doses of chemotherapeutics in human beings	
Second step: Full text	At least one conversion factor for converting the dose of a substance into the dose of another one was explicitly stated in the paper or was deductible from the doses of the respective substances.	Generally assuming equality of effect (factor = 1) without presenting supporting evidence
	The respective doses were intended for the same mode of application	Article only referenced a factor from another earlier publication unchanged and the factor could be verified in the referenced paper (except for literature reviews resulting in a definitive factor)

Articles were included if they had been published since 1985 because of incomplete availability of older publications. Inclusion of adults in the respective studies was no exclusion criterion, as we were generally not interested in the respective study results, but in the method sections. The first author screened the titles and abstracts and evaluated the full texts of the remaining articles.

As we had started with generally researching the topic of carcinogenic effects of chemotherapy in children when preparing the STATT study, literature previously available to us, which was not found by the formal literature search, was added to the review.

A conversion factor is here defined as a factor the dose of a specific chemotherapeutic drug is multiplied with to obtain the equivalent dose of the reference drug in the respective substance class. We extracted or calculated these factors based on our literature search for the substances mentioned. If necessary, units were harmonized before calculating the conversion factors. Additionally, we extracted general information on the article in which the respective factor had been used (study design and study objective, study population, time period, age group and study size).

If available in the literature, we recorded the basis for the equivalence (such as equipotency, hematotoxicity or cardiotoxicity) and the evidence behind it. The term equivalence usually refers to the treatment effect or to different kinds of toxicity.

For each substance group, the reference drug was chosen based on what was usually used in the literature. If a publication used a different reference drug, we recalculated the respective conversion factors accordingly. For glucocorticoids, prednisone was used as reference drug. Hydrocortisone‐equivalents were recalculated into prednisone‐equivalents by using a conversion factor of 0.25 according to the ‘Arzneimittelkommission der Deutschen Apotheker’,^15^ which means that a hydrocortisone dose of 1 mg/m^2^ is equivalent to a prednisone dose of 0.25 mg/m^2^. For anthracyclines, daunorubicin‐equivalents were considered equal to doxorubicin‐equivalents if only daunorubicin‐equivalents were available.

Wherever we found more than one conversion factor based on the same underlying principle for the same substance, we needed to select a factor for our purpose. We applied the following criteria (*defined* a priori) in this order: (1) most recent publication year and (2) articles which developed their own conversion factor based on their own literature review of equivalence. We present all factors found, indicating the one we selected (see section 3).

### 2.3 Conversion factors based on typical doses

This simple approach assumes that the ratio of typical doses of two substances in a group probably comes close to a conversion factor based on therapeutical equipotency.

For deriving the typical doses per substance we used a comprehensive list of all treatment protocols used in German pediatric oncology from the years 1970 to 2018,^11^, ^12^ which included 97 protocols with 678 treatment arms. Only doses given in mg/m^2^ were included (except for asparaginase, where only IU (International Units)/m^2^ were considered). The list contains cumulative doses per therapy block for each individual substance, each therapy arm and each protocol. This mainly excludes doses given in mg/kg. As a typical dose for a substance, we consider the mode, that is, the most frequently used dosage, for all doses across all therapy blocks and therapy arms of all protocols where that substance had been used. The substance Methotrexate, that is, has been used in 1515 therapy blocks over all treatment protocols and treatment arms with cumulative dosages from 12.5 to 24 000 mg/m^2^. Three hundred and seventy‐six out of these 1515 cumulative doses, and thus the most frequently used dosage (=mode), were 1000 mg/m^2^. The median dose was also 1000 mg/m^2^.

Dividing the typical doses of a reference substance and a substance yields the alternative conversion factor.

The literature search did not provide conversion factors for all substances needed in our project. In order to decide whether we can justify filling the gaps with the typical dose‐approach, we compared them using Pearson correlation coefficients and linear regressions on a log scale for all substances where factors from different approaches were available. We provide regression coefficients with confidence limits for the individual (CLI) values and the mean predicted values (CLM). The statistical analyses were performed with SAS 9.4 (proc corr and proc reg).

## 3 RESULTS

### 3.1 Literature review

**FIGURE 1:**
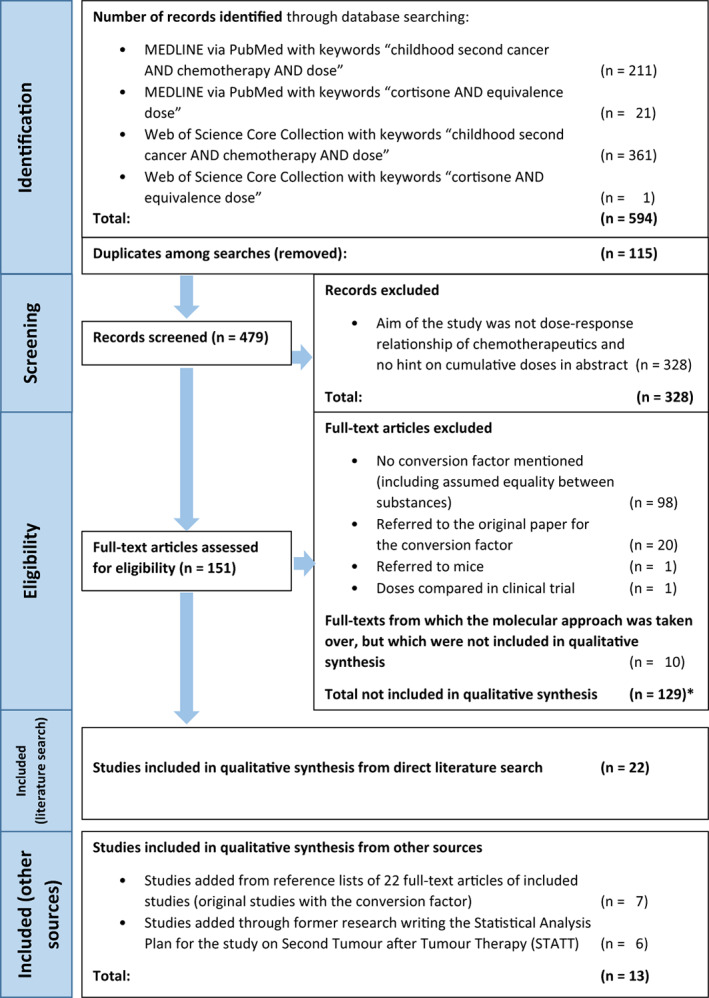
Flow chart of inclusion and exclusion of identified articles. *One study is mentioned twice because it referred to molecular weights and in a second analysis assumed equality between substances

Figure 1 gives an overview of the article selection process using the above mentioned search strategy and inclusion and exclusion criteria. In total, we identified 479 articles after removing duplicates.

The articles were rather diverse regarding the study designs and the study populations. Most of them included children or adolescents with cancer or childhood/adolescent cancer survivors and referred to study populations in Europe or North America. Except for three articles,^8^, ^16^, ^17^ the information we sought was mostly part of the methods section of the respective article as the articles were not explicitly about the factors as such.

In 10 out of the 151 articles screened which met all inclusion criteria, the authors suggested converting mg/m^2^ of chemotherapeutics to moles/m^2^ to quantify the total dose of a drug in each drug class (equimolar approach).^18^, ^19^, ^20^, ^21^, ^22^, ^23^, ^24^, ^25^, ^26^, ^27^

As molecular weights are easily available for almost all chemotherapeutic substances, we were able to calculate additional factors using this approach ourselves. The factors were calculated using the molecular weights, independently from any article. The higher the molecular weight, the fewer active molecules are included per weight of a substance. Under this assumption, we calculated factors derived from the molecular weights for each substance as described above for the other factors.

Twenty‐two further studies not (only) using the equimolar approach met all inclusion criteria. Additionally, we identified seven more articles of this type, which had been cited by the articles identified in the original search,^16^, ^17^, ^28^, ^29^, ^30^, ^31^, ^32^ and added another six articles which had been known from our former general research^5^, ^9^, ^10^, ^15^, ^33^, ^34^ on late effects of childhood cancer. Hence, 35 articles with conversion factor suggestions other than those based on the equimolar approach were included in the literature review. Tables 2 and 3 list all 24 studies examining chemotherapeutics other than glucocorticoids (Table 2) and 11 articles examining glucocorticoids (Table 3) separately.

**TABLE 2:** Overview of 24 articles included in the literature review examining chemotherapeutics other than glucocorticoids^a^: summary of study characteristics, study design and stated basis for equivalence

Article	Study population	Date of recruitment	Children/Adults^b^	Country	Number of patients	Study design	Study objective	Stated basis for equivalence and respective reference (only references since the year 1985)	Conversion factors provided for	Remark
Allodji (2021)^6^	Childhood cancer survivors; Cases: secondary leukemia	1930–2000	Children at diagnosis	France, United Kingdom, United States, Canada, Italy, and the Netherlands	147 cases/522 controls	Pooled data from four nested case–control studies	Risk of secondary leukemia in relation to chemotherapy	Hematological toxicity based on Green et al. 2014^8^	AA	Additional factor for dacarbazine in article
Blanco (2012)^33^	Childhood cancer patients treated at member organization of Children's Oncology Group (COG); Cases: with additional cardiomyopathy	1966–2008	Children and adolescents ≤21 years at diagnosis	Several	170 cases/317 controls	Case–control study	Dose‐dependent risk of anthracycline‐related cardiomyopathy	Cardiotoxicity based on Lehmann et al. 2000^32^	A	Supplement of Blanco 2012^33^ presents factors different from Lehmann 2000^32^
Casagranda (2016)^7^	Cohort of Childhood Cancer Registry Rhônes‐Alpes Region with all types of first and second primary	1987–2004 (diagnosis of first primary)	Children	France	64 cases/190 controls	Nested case–control‐study	Secondary neoplasia comparing chemotherapy from different groups	“hematologic toxicity or rules of substitution” based on Guérin 2003^23^ and Tucker 1987^35^	AA, A, E, PD	Factors presented in article are identical to Guérin 2007,^3^ not to Guérin 2003^23^
Children's Oncology Group (2018)^10^	Childhood Cancer Survivors	–	Children and adolescents	–	–	Guideline developed from a literature review	–	Cardiotoxicity based on literature review for this guideline	A	–
Creutzig (2007)^36^	Children with primary or secondary AML	1993–2003 (treatment)	Children	Germany	1207	Follow‐up after clinical trial	Cardiotoxicity	Cardiotoxicity (not explicitly stated, assumed due to outcome of the study) based on^37^, ^38^, ^39^	A	References in article not mentioning a definitive factor^37^, ^38^, ^39^
Feig (1996)^40^	Children with acute lymphatic leukemia at first bone marrow relapse from Children's Cancer Group (CCG)	1990–1992	Children and adolescents <21 years at diagnosis	USA, Canada	92	Randomized Clinical Trial	Event‐free survival	“Isodose conversion factor” based on Berman 1991^41^ and Wiernik 1992^42^	A	Conversion factors used in references of the article were slightly different from those used in article itself; references in article refer to clinical trials comparing idarubicin versus daunorubicin— > references not included additionally
Feijen (2015)^30^	Childhood cancer survivors	1962–2002	Children and adolescents <25 years at diagnosis	Several	15 815	Cohort studies	Hazard Ratio for cardiotoxicity (Heart failure) comparing Daunorubicin to Doxorubicin	Cardiotoxicity assumed based on references stated in introduction	A	Conversion factors concluded from calculated hazard ratio not included
Feijen (2019)^34^	Childhood cancer survivors	1962–2005	Children and adolescents <23 years at diagnosis	Several	28 423	Cohort studies	Hazard Ratio for cardiotoxicity (Heart failure) comparing different anthracyclines	Cardiotoxicity assumed based on references stated in introduction	A	Conversion factors concluded from calculated hazard ratio not included
Green (2014)^8^	Literature review	–	Not stated	Several	Literature review	Literature review	Literature review on hematological toxicity, comparison of Alkylating Agent Dose (AAD) with developed CED (cyclophosphamide equivalent dose)	Hematological toxicity based on literature review in article	AA	–
Guerin (2007)^3^	Childhood cancer survivors from solid tumor	1942–1986	Children at diagnosis	France, UK	153 cases/442 controls	Nested case–control study in a European cohort	Risk of secondary neoplasia in relation to chemotherapy	“Hematological toxicity or substitution rules” based on Le Deley 2003^4^	AA, A, E, PD, VA	Four additional factors not mentioned in Le Deley 2003,^4^ but in Guerin 2007^3^
Henderson (2012)^43^	Childhood cancer survivors with (cases) or without (controls) secondary sarcomas	1970–1986 (first diagnosis), 2000 (baseline)	Children and adolescents <21 years at diagnosis	USA, Canada	105 cases/422 controls	Nested case–control study in the Childhood Cancer Survivor Study	Secondary sarcoma comparing chemotherapy from different groups	Not stated	A, E, PD	–
Launchbury (1993)^17^	Literature review	–	Not stated	Several	Literature review	Literature review	Comparison of characteristics, therapeutic activity and toxicity of epirubicin and daunorubicin	Antitumour efficacy based on literature review in the article	A	–
Le Deley (2003)^4^	Childhood cancer survivors of a solid tumor with (cases) or without (controls) secondary leukemia	1980 (first neoplasia), secondary neoplasia up to 1999	Children at diagnosis	France	61 cases/196 controls	Case–control study	Secondary leukemia comparing chemotherapy from different groups	“Hematologic toxicity or substitution rules”; reference not stated	AA, A, E, PD	–
Lehmann (2000)^32^	Consecutive patients with AML, ALL, CML, multiple myeloma or breast cancer and hematopoetic stem cell transplantation	1985–1994	Adolescents and Adults between 17 and 62 years old	Sweden	148	Case series	Cardiac systolic function with anthracycline dose as risk factor	“Equipotency of anthracycline doses and the cardiotoxic potential of the drugs” based on two references after the year 1985	A	References in the article are literature reviews without a definitive factor^44^, ^45^
Messinger (1999)^46^	Patients with acute lymphoblastic leukemia	–	Children and adolescents up to 21 years (most studies)	Several	Literature review	Literature review	Benefit of anthracyclines	Equipotency assumed due to reference to 17	A	Reference 17 in the article contains only factor for epirubicin
Mouridsen (1990)^16^	Patients with breast cancer	–	Adults (not stated, but most likely because of breast cancer)	Literature review	Literature review	Review of phase II and phase III trials	Efficacy of anthracyclines (comparison of response rates)	Hematological and non‐hematological toxicity and cardiotoxicity based on review performed in the article	A	–
Mulrooney (2009)^28^	Survivors of childhood cancer in the Childhood Cancer Survivor Study (CCSS)	1970–1986 (enrolment), 2000–2002 (follow‐up)	Children and adolescents <21 years at diagnosis	USA	14 358 survivors and 3899 siblings	Retrospective cohort study	Cardiac outcomes	Basis for equivalence not stated; factors based on Pai 2000^47^	A	Reference in the article is review without explicit factors^47^
Mulrooney (2016)^5^	Survivors of childhood cancer in the St. Jude Lifetime Cohort Study	2013 (follow‐up)	Children (“childhood cancer”) at diagnosis	USA	1853	Cross‐sectional study	Cardiotoxicity	“Isotoxic equivalents” based on Le Deley 2003^4^: “hematologic toxicity or substitution rules”	A	Idarubicin mentioned in Mulrooney 2016,^5^ but not in Le Deley 2003^4^
Ozols (1985)^31^	Advanced ovarian cancer patients refractory to standard therapy	–	Adults (assumed)	USA (assumed)	Several clinical trials	Several clinical trials and in‐vitro studies comparing high dose cisplatin and high dose carboplatin	Clinical activity and toxicity, in vitro and clinical trial	“Clinically active” based on literature before 1985	PD	–
Schramm (2019)^9^	Children with acute lymphatic leukemia	2003–2010 (inclusion)	Children	Germany	773	Clinical trial	Survival (Overall Survival, Event‐free survival)	Not stated	Asp	–
Smibert (2004)^29^	Childhood cancer patients with anthracycline exposure more than 12 month after anthracycline treatment; controls from healthy siblings and patient peers	1977–1995 (diagnosis)	Children at diagnosis	Australia (authors)	110 cases/31 controls	Cross‐sectional, non‐randomized study	Cardiac outcome of different doses of anthracyclines and alkylating agents	Anthracyclines: “Myelosuppressive potency”; no reference stated	A	–
Sorensen (2003)^48^	Acute lymphoblastic leukemia survivors and Wilms tumor survivors	1970–1990 (treatment of Wilms Tumor)	Children at diagnosis	UK	101 acute lymphoblastic leukemia survivors, 83 Wilms tumor survivors and 100 controls	Prospective, longitudinal study	Cardiac toxicity of different doses of anthracyclines	Cardiac toxicity based on the assumption of equivalence of cardiac toxicity between daunorubicin and doxorubicin	A	–
Wang (2022)^49^	Childhood Cancer survivors included in seven cohorts	1946–2012 (diagnosis)—2021 (follow‐up)	Children and adolescents <21 years at diagnosis	Europe, North America	21 892	Pooled Individual patient data cohort	Subsequent breast cancer	Hematological toxicity assumed due to reference to cyclophosphamide equivalent dose; factors based on cyclophosphamide equivalent dose	AA	–
Winick (1993)^50^	Patients with acute lymphoblastic leukemia treated with etoposide	1986–1991 (diagnosis)	Children	USA	205	Follow‐up of consecutive patients	Event‐free survival, secondary AML, no comparison group	Not stated; factors based on literature before 1985	E	–

Abbreviations: A, anthracyclines; AA, alkylating agents; ALL, acute lymphatic leukemia; AML, acute myelocytic leukemia; Asp, asparaginase; CML, chronic myelocytic leukemia; E, epipodophyllotoxins; PD, platinum derivates; VA, vinca alkaloids.

^a^ Articles which (1) mentioned a conversion factor or doses which permitted the calculation of a conversion factor, (2) have been published since the year 1985 and (3) of which a full‐text in English or German was available (see Table 1).

^b^ In studies on childhood cancer survivors, the inclusion in the study might have taken place as adults. The age groups were defined as follows: children: below the age of 18; adults: above the age of 18; adolescents: age 16–25 (only mentioned if there were mixed groups of either adolescents and adults or children and adolescents).

**TABLE 3:** Overview of 11 articles included in the literature review examining glucocorticoids^a^: summary of study characteristics, study design and stated basis for equivalence

Article	Study population	Date of recruitment	Children/adults^b^	Country	Number of patients	Study design	Study objective	Stated basis for equivalence and respective reference (only references since the year 1985)	Remark
Arteaga (1999)^51^	Patients with persistent hypokalemia after successful adrenalectomy due to Cushing's syndrome due to ectopic ACTH secretion	Not stated	Adults	Chile	1	Case report	Hypokalaemia	Not stated	–
Arzneimittelkommission der Deutschen Apotheker^15^	–	–	Adults	Germany	–	Tables of equivalent doses	–	Antiinflammatory potency based on literature research of the “Arzneimittelkommission der Deutschen apotheker”	–
Bostrom (2003)^52^	Patients with acute lymphoblastic leukemia (ALL) in the Children's Cancer Group	1993–1995	Children	USA, Canada	1060	Randomized controlled trial (2 × 2 factorial design) comparing dexamethasone versus prednisone	Relapse and event free survival	“Dexamethasone is approximately 7‐fold more potent than prednisone”; statement based on “published equivalency tables” from in‐vitro studies	–
Ekstrand (2020)^53^	Patients with hypopituitarism receiving growth hormone replacement	1990–2002	Adults	Sweden (authors)	229	Prospective trial (post‐hoc analysis): 1 switch group (cortisone acetate— > hydrocortison), two control groups (cortisone acetate only and no glucocorticoid replacement)	Metabolic effects	Antiinflammatory potency based on Filipsson 2006^54^ and on literature from before 1985	–
Filipsson (2006)^54^	Hypopituitary patients from KIMS (Pfizer International Metabolic Database)	2004 (inclusion)	Adults	28 countries in database, non‐European patients excluded	2424	Longitudinal survey, examination at baseline and one year after growth hormone treatment	Metabolic outcome comparing three groups (hydrocortisone, cortisone acetate, and prednisolone/dexamethasone)	”Previous antiinflammatory comparisons” based on references from before 1985	–
Lovas (2006)^55^	Patients with Addison's disease and healthy controls	Not stated	Adults	Norway	31 patients with Addison's disease and 20 healthy controls	Correlational study	Correlation of serum and saliva cortisol	“Conventional glucocorticoid replacement therapy” based on Arlt 2003^56^	Reference in article^56^ is literature review without definitive factor
Pfeiffer (1992)^57^	Patients with avascular osteonecrosis of the femoral head after steroid therapy for cerebral trauma	1981–1987 (accident)	Adolescents	Germany	3	Case series	Avascular osteonecrosis of the femoral head	“prednisone equivalent” based on textbooks^58^, ^59^	–
Puglisi (2021)^60^	Patients with an established diagnosis of adrenal insufficiency	1995–2018	Adults	Italy	203	Case series	Influence of the etiology of adrenal insufficiency on the types of glucocorticoid used	Hydrocortisone equivalent dose (HEC); no reference stated	–
Sandrini (1993)^61^	Patients with salt‐losing form of congenital adrenal hyperplasia due to 21‐hydroxylase deficiency	Not stated	Children and adolescents	USA	19	Case series	Variation of cortisol dose with age	“Equivalent dose of oral cortisol” based on two articles before 1985 and two textbooks^62^, ^63^	–
Swords (2003)^64^	Hypopituitary patients	Not stated	Adults	UK (authors)	10	Prospective, cross‐over study	Influence on growth hormone therapy comparing different glucocorticoids	Not stated	–
Tabone (2021)^65^	Childhood cancer survivors from acute leukemia included in follow‐programme LEA and fulfilling certain criteria	Since 1980	Children at diagnosis, Adults at follow‐up	France	89	Follow‐up of a cohort	Factors influencing bone mineral density	Not stated	–

Abbreviations: ALL, acute lymphatic leukemia; AML, acute myelocytic leukemia; CML, chronic myelocytic leukemia.

^a^ Articles which (1) mentioned a conversion factor or doses which permitted the calculation of a conversion factor, (2) have been published since the year 1985 and (3) of which a full‐text in English or German was available (see Table 1).

^b^ In studies on childhood cancer survivors, the inclusion in the study might have taken place as adults. The age groups were defined as follows: children: below the age of 18; adults: above the age of 18; adolescents: age 16–25 (only mentioned if there were mixed groups of either adolescents and adults or children and adolescents).

Two^30^, ^34^ out of these 35 articles set out to challenge the idea that factors based originally on hematologic toxicity can be used for studying cardiologic late effects, citing a large number of such factors previously used. We extracted these factors stated in the methods section of the articles according to our criteria in Table 1. As the same literature was cited in both articles, we only included the factors derived from the literature and used in the study in the later article.^34^

### 3.2 Principles for effect equivalence—chemotherapeutics other than glucocorticoids

The basis of assessment of the different principles for effect equivalence other than the equimolar approach was usually not entirely clearly stated and rather diverse. For chemotherapeutics except glucocorticoids (24 articles), the conversion factors in the literature were mostly (15 out of 24) based on a principle which can be summarized by the term isotoxic. Toxicity referred to cardiotoxicity (*n* = 6), hematotoxicity (*n* = 8), or hematological toxicity, non‐hematological toxicity and cardiotoxicity (*n* = 1). An isotoxicity factor of, for example, four for a substance means that one unit of the substance was considered four times more toxic than one unit of the reference substance.

Three articles (3 out of 24) referred to the intended effects of the chemotherapeutics, using the terms antitumor efficacy (*n* = 2) or potency (*n* = 1), respectively. These can be summarized by the term equipotency. One additional article justified a factor with both cardiotoxicity (isotoxicity) and potency (equipotency).

Five articles out of 24 did not explicitly state a basis for their conversion factors; we conclude an underlying assumption of isotoxocity or equipotency from the context and usage of the factors in the respective studies.

For 17 substances, more than one factor was found in the literature. For all substances except Thiotepa, these factors were generally rather similar; however, the basis stated could still differ. As an example: Epirubicin was presented with a factor of 0.67 based on hematological toxicity^4^, ^5^, ^7^ as well as on cardiotoxicity.^10^, ^33^ Another article mentioned similar factors for epirubicin based on hematological toxicity, cardiotoxicity or non‐hematological toxicity, respectively.^16^ One article justified the factors for anthracyclines with both cardiotoxicity (isotoxicity) and potency (equipotency).^32^

### 3.3 Principles for effect equivalence—glucocorticoids

For glucocorticoids (11 articles), all factors in the literature were based on the concept of equipotency (*n* = 8). In these articles, the following principles were used: potency (either general or inflammatory) (*n* = 4), conventional glucocorticoid replacement therapy (*n* = 1), hydrocortisone‐equivalent dose (*n* = 2) or prednisone equivalent (*n* = 1). In three articles, the basis of the conversion factor was not stated explicitly. We conclude an underlying assumption of equipotency from the context and usage of the factors in the respective study.

The usage of and stated bases for conversion factors in the literature seem to suggest that at least some authors assume the concepts of isotoxicity, isotoxicity for a specific outcome, and equipotency are sufficiently similar for general usage in late effects research. We concur for now and will refer to both concepts (isotoxicity and equipotency) as effect equivalence below. These factors are listed in Table 4 column 3.

**TABLE 4:** List of substances used in pediatric oncology with respective conversion factors to convert the dose of a substance into the dose of the respective reference substance

Anatomical therapeutic chemical (ATC) Code^2^	Drug	Different types of conversion factors						
		Factors based on effect equivalence from literature review			Novel approach: factors considering mode of all doses in respective German trials^11^, ^12^		Factors from molecular weight of the respective substances	
		Conversion factor to reference drug	Reference	Preferred^a^	Typical Dose (mg/m^2^)	Conversion factor to reference drug	Molecular weight (g/mol)	Conversion factor to reference drug
**Corticosteroids (H02AB)**								
H02AB02	Dexamethasone	7.25	15	Yes	100.0	18.40	392.50	0.91
		7.70	54	–				
		6.67	52, 65	–				
		10.00	51	–				
		7.5	57	–				
H02AB04	Methylprednisolone	1.25	15	Yes	–	–	374.50	0.96
H02AB06	Prednisolone	1.00	15, 54	Yes	300.0	6.10	360.40	0.99
**H02AB07**	**Prednisone**	**1.00**	**reference drug**	**Yes**	**1837.5**	**1.00**	**358.40**	**1.00**
H02AB09	Hydrocortisone	0.25	15, 54, 61	Yes	–	–	362.50	0.99
H02AB10	Cortisone	0.20	15, 53, 54, 55, 60, 64	Yes	–	–	360.50	0.99
		0.21	61	–				
**Alkylating agents (L01A)**								
**L01AA01**	**Cyclophosphamide**	**1.00**	**reference drug**	**Yes**	**1000.0**	**1.00**	**261.08**	**1.00**
L01AA02	Chlorambucil	14.29	6, 8, 49	Yes	–	–	304.20	0.85
		10.00	3	–				
L01AA03	Melphalan	40.00	6, 8, 49	Yes	140.0	7.14	305.20	0.85
		43.00	3, 4, 7	–				
L01AA05	Chlormethine	83.30	3, 4, 7	–	–	–	156.05	1.67
		100.00	6, 8, 49	Yes				
L01AA06	Ifosfamide	0.24	6, 8, 49	Yes	6000.0	0.17	261.08	1.00
		0.25	3, 4, 7	–				
L01AA07	Trofosfamide	–	–	–	150.0	6.67	323.58	0.81
L01AB01	Busulfan	8.82	6, 8, 49	Yes	600.0	1.67	246.30	1.06
		10.00	3, 4, 7	–				
L01AB02	Treosulfan	–	–	–	12000.0	0.08	278.30	0.93
L01AC01	Thiotepa	50.00	6, 8, 49	Yes	30.0	33.30	189.22	1.37
		6.67	3, 4, 7	–				
L01AD01	Carmustine	15.00	6, 8, 49	Yes	–	–	214.50	1.22
		10.00	3, 4, 7	–				
L01AD02	Lomustine	16.00	6, 8, 49	Yes	600.0	1.67	233.69	1.11
		10.00	3, 4, 7	–				
L01AX03	Temozolomide	–	–	–	3150.0	0.32	194.15	1.35
L01AX04	Dacarbazine	2.00	3, 4, 7	–	750.0	1.33	182.18	1.43
		3.77	6	Yes				
L01XB01^b^	Procarbazine	0.86	6, 8, 49	Yes	3000.0	0.33	221.30	1.18
		1.00	3, 4, 7	–				
L01XX11^c^	Estramustine	0.15	3	Yes	–	–	440.40	0.59
**Antimetabolites (folic acid analogues) L01BA**								
**L01BA01**	**Methotrexate**	–	–	–	**1000.0**	**1.00**	**454.40**	**1.00**
**Antimetabolites (purine analogues) L01BB**								
**L01BB02**	**Mercaptopurine**	–	–	–	**525.0**	**1.00**	**152.18**	**1.00**
L01BB03	Thioguanine	–	–	–	500.0	1.10	167.19	0.91
L01BB04	Cladribine	–	–	–	12.0	43.80	285.69	0.53
L01BB05	Fludarabine	–	–	–	150.0	3.50	365.21	0.42
L01BB06	Clofarabine	–	–	–	200.0	2.63	303.68	0.50
**Antimetabolites (pyrimidine analogues) L01BC**								
**L01BC01**	**Cytarabine**	–	–	–	**600.0**	**1.00**	**243.22**	**1.00**
L01BC02	Fluorouracil	–	–	–	3600.0	0.17	130.08	1.89
**Vinca alkaloids L01CA**								
L01CA01	Vinblastine	0.25	3	Yes	12.0	0.13	811.00	1.02
**L01CA02**	**Vincristine**	**1.00**	**reference drug**	**Yes**	**1.5**	**1.00**	**825.00**	**1.00**
L01CA03	Vindesine	0.50	3	Yes	3.0	0.50	753.90	1.09
**Epipodophyllotoxins L01CB**								
**L01CB01**	**Etoposide (VP‐16)**	**1.00**	**reference drug**	**Yes**	**450.0**	**1.00**	**588.60**	**1.00**
L01CB02	Teniposide	1.00	3, 4, 7, 43	Yes	165.0	2.73	656.70	0.89
		2.00	50	–				
**Topoisomerase inhibitors (other than epipodophyllotoxines**) **L01CE**								
**L01CE01**	**Topotecan**	–	**reference drug**	–	**7.0**	**1.00**	**457.91**	**1.00**
**Antibiotics except anthracyclines L01D**								
**L01DA01**	**Dactinomycin**	–	**reference drug**	–	**1.5**	**1.00**	**1255.40**	**1.00**
L01DC01	Bleomycin	–	–	–	30.0	0.05	1415.60	0.88
L01DC03	Mitomycin	–	–	–	8.0	0.19	334.30	3.70
**Anthracyclines L01DB**								
**L01DB01**	**Doxorubicin**	**1.00**	**reference drug**	**Yes**	**60.0**	**1.00**	**543.50**	**1.00**
L01DB02	Daunorubicin	1.00	28, 34, 36, 43, 48	Yes	120.0	0.50	527.50	1.03
		0.83	3, 4, 5, 7, 33	–				
		0.75	32, 46	–				
		0.67	29	–				
		0.50	10	–				
L01DB03	Epirubicin	1.00	17, 34, 46	Yes	150.0	0.40	543.50	1.00
		0.67	4, 5, 7, 10, 33	–				
		0.75	32	–				
		0.83^d^ 0.67^e^ 0.56^f^	16	–				
L01DB04	Aclarubicine	–	–	–	125.0	0.48	811.88	0.67
L01DB05	Zorubicine	0.50	4, 7	Yes	–	–	645.7	0.84
L01DB06	Idarubicin	5.00	5, 10, 33, 34, 36	Yes	14.0	4.29	497.50	1.09
		3.00	28, 32, 43	–				
		2.78	7	–				
		4.50	40	–				
L01DB07	Mitoxantrone	4.00	4, 5, 7, 10, 34	Yes	20.0	3.00	444.50	1.22
		3.00	32	–				
		5.00	36	–				
**Platinum derivates L01XA**								
**L01XA01**	**Cisplatin**	**1.00**	**reference drug**	**Yes**	**100.0**	**1.00**	**300.00**	**1.00**
L01XA02	Carboplatin	0.25	3, 4, 7, 31, 43	Yes	600.0	0.17	371.25	0.81
**Asparaginase L01XX**								
**L01XX02**	**Asparaginase**	**1.00**	**reference drug**	**Yes**	**25000.0** ^g^	**1.00**	**–**	**–**
L01XX24	Pegylated asparaginase	18.00	9	Yes	2500.0^g^	10.00	**–**	**–**

*Note*: Reference substances are printed in bold.

^a^ Based on two criteria (*defined* a priori), which were applied in the following order: (1) most recent publication year, (2) articles which developed their own conversion factor based on their own literature review.

^b^ According to the ATC index, Procarbazine is a Methylhydrazine (L01XB) and belongs to the group “other antineoplastic agents” (L01X). However, due to its mode of action, it is usually grouped with the alkylating agents (L01A) in oncology literature.^3^, ^8^

^c^ According to the ATC index, Estramustine belongs to the group “other antineoplastic agents” (L01X). However, due to its mode of action, it is usually grouped with the alkylating agents (L01A) in oncology literature.^3^, ^8^

^d^ Hematological toxicity.

^e^ Non‐hematological toxicity.

^f^ Cardiac toxicity.

^g^ IU (International Units)/m^2^.

### 3.4 Equimolar principle

The rationale behind the equimolar principle is that ‘a molecule of a given drug generally has one active site, whatever its weight. Even if a particular drug may have more than one active site per molecule, the error introduced by this hypothesis is probably lower than that introduced when summing the weights’.^27^ The molecular weights (g/mol) of substances with an ATC code are readily available for all substances from the Website PubChem,^66^ not only for the substances included in the papers found in the literature search.^18^, ^19^, ^20^, ^21^, ^22^, ^23^, ^24^, ^25^, ^26^, ^27^

This permitted directly calculating equimolar conversion factors for all substances (save one, see below), presented in column 9 in Table 4.

For asparaginase (ATC‐code L01XX02) and pegylated asparaginase (L01XX24), we could not present factors derived from molecular weights because this chemical approach is not applicable to enzymes.

### 3.5 Conversion factors based on typical dose

Typical doses were available for 41 (of the 49) substances, including the 11 substances for which no conversion factor based on effect equivalence had been found in the literature. The remaining eight (49 minus 41) substances have not been used in treatment protocols in German pediatric oncology since the 1970s. The resulting conversion factors are presented in Table 4, column 7.

### 3.6 Comparing factors based on different principles

Table 4 presents the conversion factors for each substance by substance group for the principles of assumed effect equivalence (as found in the literature), based on typical dose,^11^, ^12^ and based on the equimolar principle.^66^

The conversion factors based on effect equivalence derived from the literature ranged from 0.15 to 100, those derived from typical doses ranged from 0.05 to 43.8. More than 80% or 90%, respectively, of these conversion factors were between 0.1 and 10. The range of the factors based on molecular weights, 0.42–3.70, was much narrower.

**FIGURE 2:**
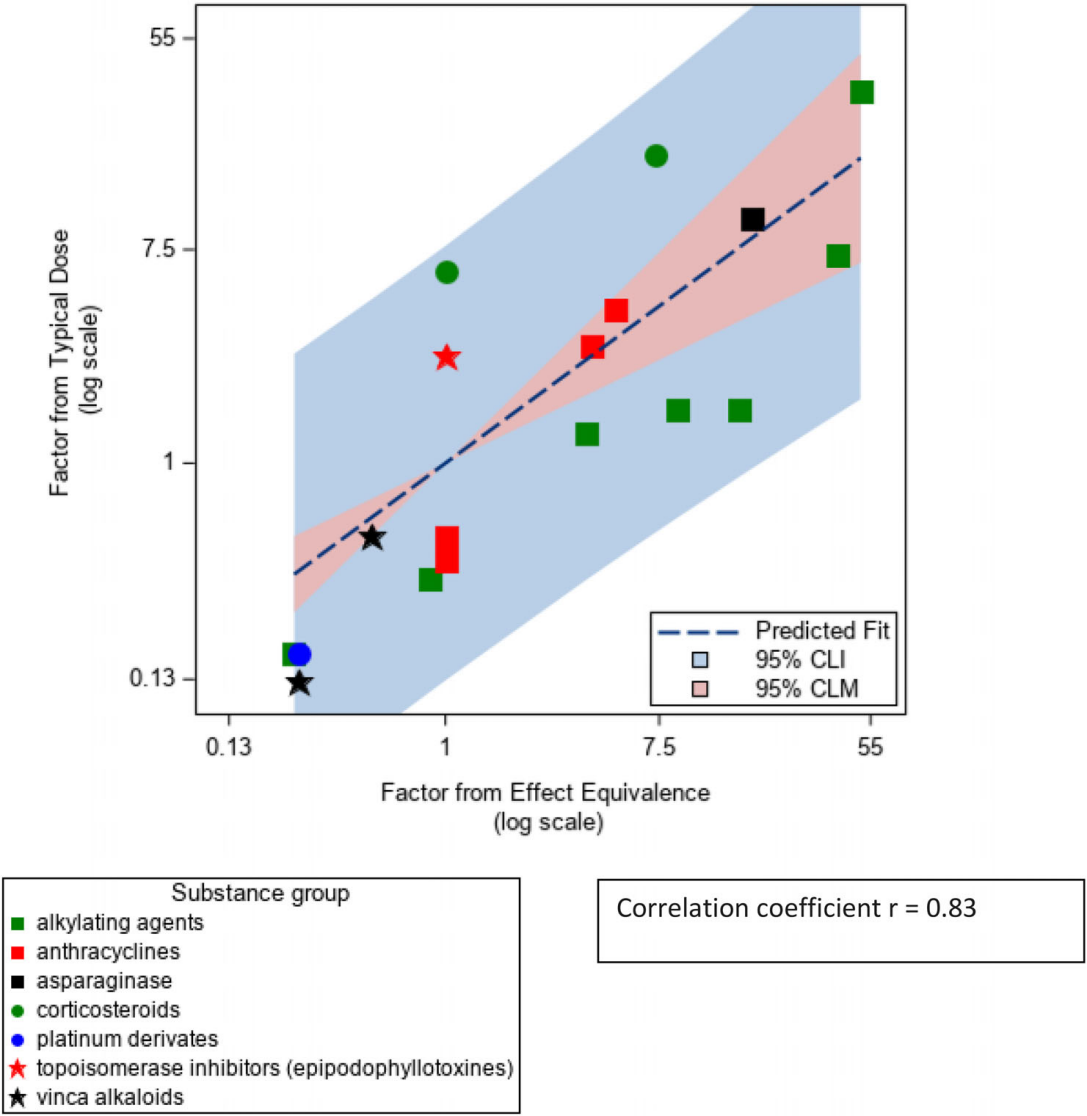
Scatter plot and regression line of the factors based on effect equivalence from literature review and the factors based on typical dose (derived from treatment protocols in pediatric oncology in Germany). CLI, confidence limits for the individual predicted values; CLM, confidence limits for the mean predicted values

Comparing the factors, the correlation closest to 1 was found between the factors based on the principle of effect equivalence and the typical dose principle, *r* = 0.83. Figure 2 shows the corresponding scatter plot (factors on a log scale). The slope from the linear regression model was 0.74, so the factors from typical dose were on average closer to one than the ones suggested in the literature based on effect equivalence. A slope of 1 would mean factors from both principles would be fully comparable on average. Sensitivity analyses and subgroups are presented in Supplementary Figures 1 and 2. Results differed slightly when excluding glucocorticoids (Supplementary Figure 2).

The correlations of the factors based on the equimolar principle with the other factors were considerably lower (*r* = 0.54 equimolar versus the principle of effect equivalence, and −0.32 equimolar compared to factors based on the typical doses). Supplementary Figures 3 and 4 present the corresponding scatterplots.

## 4 DISCUSSION

In epidemiological research on late effects of the therapy of childhood cancer, it can be necessary to aggregate chemotherapy agents into substance classes, as in our study on second tumors after tumor therapy (STATT). We started out by considering aggregating drugs without using any conversion inappropriate because it is important to adjust for the different potencies or toxicities of the drugs, as stated in the literature, for example References 4, 8. Additionally, results obtained from aggregating doses without conversion are not transferable to other studies with another mix of drugs.

According to the criteria from Munn et al.^67^ a scoping review seemed appropriate for our research aim to identify conversion factors for chemotherapeutic substance classes used in literature.

In a literature search, 35 articles were identified which used or justified conversion factors for 26 substances (excluding the 12 reference substances) based on principles which can be summarized as effect equivalence. The literature review did not yield such conversion factors for 11 relevant chemotherapeutic substances used in treatment protocols in Germany. Further 10 papers suggested the equimolar principle using molecular weights.^18^, ^19^, ^20^, ^21^, ^22^, ^23^, ^24^, ^25^, ^26^, ^27^ For 41 substances we were able to derive a factor based on a typical dose approach based on a comprehensive list of substances used in German pediatric oncology.

Comparing these three types of conversion factors, we found the effect equivalence‐based and the typical dose‐based factors to be highly correlated (*r* = 0.83) and on average close to being identical. The correlation of the factors derived from molecular weights with the other factors was moderate or close to zero.

The literature search was not straightforward, as only three articles^8^, ^16^, ^17^ were explicitly about the factors as such. All other articles mentioned the factors briefly in their respective methods sections. In a second step we searched the reference lists of the articles found by literature search in order to identify the original source for the factor mentioned in them. If the factor was the same, the original article was included instead of the article found by literature search. The search on glucocorticoids could not be restricted to second tumors after childhood cancer as we found only 22 articles with our broad search on equivalence dose for glucocorticoids.

The literature search was rather broad in scope to ensure we would not miss any relevant papers. However, the fact that we identified 13 additional papers from reference lists or through prior knowledge indicates that there were potential blind spots. These 13 articles applied substance conversion outside the topic we were primarily interested in (second tumors after childhood cancer, *n* = 11) or were not listed in PubMed or Web of Science as they were a guideline^10^ and a table of equivalent doses^15^ (*n* = 2).

The typical doses‐approach was feasible and had a very broad information basis, as pediatric oncology in Germany has been using nationwide, centralized treatment protocols since the 1970s,^68^ and we had access to a complete overview over all these protocols until 2018.^11^, ^12^

We needed a criterion to select a factor when more than one was available in the literature. Using the latest information and one which stated its basis clearly seemed sensible, but they are still somewhat arbitrary and readers may make a different selection from Table 4. As factors are relatively similar, this does not influence results considerably. The only exception is Thiotepa (ATC code L01AC01), where the factors differ considerably and the one not originally selected (6.67^3^, ^4^, ^7^) yielded more plausible converted doses than the one we would have selected by our criteria (50^6^, ^8^, ^49^), as the larger factor created extreme outliers in the distribution of aggregated alkylating agents.

The method used by the authors of two articles^30^, ^34^ to derive factors from their own data was based on substance specific regression coefficients after applying a factor from the literature to then compare the effect sizes per dose. The authors suggest to use these ratios for obtaining a different conversion factor for a joint estimate; they do not apply this factor to obtain a joint dose–response estimate for their outcome, however. This is an interesting approach. One must be aware, however, that small studies and substances with small numbers of exposed patients are likely to randomly produce outlying regression coefficients, which could provide these substances randomly with an outlying weight (although the bootstrap approach chosen would render such estimates less likely). Moreover, it is questionable whether such sets of weights derived from one set of patients (or sets of patients) can be applied to another set of patients. Interestingly, the factors they derived from the data were not wildly different from the ones they cited from the literature (except for mitoxantrone). Nevertheless, we decided to err on the side of caution and to exclude these factors from our table and to focus on factors based on more general principles. Thus, we included the factors the authors of the articles^30^, ^34^ cited from literature. Our readers may come to a different conclusion.

The factors derived from typical doses were mainly based on the doses in single treatment blocks of a respective clinical study protocol. The cumulative doses for the whole therapy concept were not included in the compendium.^11^, ^12^ It does not contain any rules of replacement either. We calculated the mode of all doses listed for each substance in the compendium. We considered the mode, the dose which was used most frequently, to describe the typical dose best, independently from the number of chemotherapy blocks included in the compendium in which the respective substance was applied. We noted that the mode was almost always identical to (32 out of the 41 substances extracted from the compendium) or very close to the median.

The scientific basis for the various types of effect equivalence was often not stated (9 articles), did not yield a definitive factor (*n* = 4) or presented factors additional to or different from the ones cited in the original articles (*n* = 5). It is open for discussion to which extent conversion factors, specifically referring to cardiotoxic or hematotoxic side effects, are transferable to other endpoints in late effects research. No article referred explicitly to an equivalence of effect regarding second neoplasms, unless we equate hematoxicity with second leukemia. However, the literature search also showed that authors referring to different bases for their respective conversion factors nevertheless came to rather similar factors.

We are aware of further limitations. When applying the conversion factors in research, characteristics like dose frequency or the combination of drugs or drugs and other components of therapy, such as radiotherapy, may influence the effect equivalence. Substance combinations and dosages strongly depend on the cancer type. Conversion factors may possibly be age‐ or sex‐specific, which was however not mentioned in any of the papers found. There was no information in the available literature to calculate different conversion factors for different modes of application. For instance, for dexamethasone, the study^57^ referring to intravenous application used almost the same conversion factor as the other studies generally referring to oral application. No conversion factors for intrathecal application were mentioned in the literature. Bioavailability of intrathecal application is considered the same as intravenous.^69^ As to the factors from typical doses, Methotrexate was the only substance with information on intrathecal application and a dose given in mg/m^2^.^11^, ^12^ As only 22 therapy blocks were involved, neither the median nor the mode changed when including or excluding these doses. The typical dose was derived from German data only. Applying the principle to an overview of therapy protocols from another setting might yield different factors.

As we decided to use all information available to us for our own calculations, the literature review was based on a slightly different period than the typical dose‐approach (1985—November/December 2022 and 1970–2018, respectively): Besides technical reasons (incomplete availability of publications before 1985), we wanted to include the latest available literature in the literature review.

When comparing typical dose‐based factors to effect equivalence‐based factors from the literature, the slope from linear regression was 0.74; however, ideally the slope should have been even closer to 1.

It needs to be stated that all approaches described here are not meant for a clinical setting, for example, when replacing one substance with another for the treatment of an individual patient. This is also true for the typical dose approach. The protocols where the typical doses were derived from are used in clinical setting. However, the calculation of the typical doses was across all protocols and therefore all diagnoses, age groups and combination of drugs and was based on typical doses of single therapy blocks. Therefore, they need not be valid in all special clinical settings. Hence, all factors presented here are meant for and are particularly useful for population‐based epidemiological research. Practical application requires harmonizing units before applying the conversion factor. If height and weight of a patient are available, mg per kg can be converted into mg per m^2^.

Most of the literature cited here was about post‐hoc treatment assessment in a late effects research setting.

This study gives an overview over dose conversion factors of anticancer agents to a reference substance within their class by mode of action with an emphasis on usage in childhood cancer late effects research. We were able to present factors for 49 substances.

As a first step we present results from a literature review. The factors based on effect‐equivalence seem to be more widely used and well justified for late effects research. For substances for which no such conversion factors could be found in the literature, we proposed factors from a rather simple approach, relating typical doses. Our original question had been whether we could justify filling in these factors for the 11 substances where we could not find an effect‐equivalence factor in the literature. Based on our comparison results we consider this justified. The data base for the typical dose approach was specific for pediatric oncology in Germany; therefore, our factors may not be directly applicable to adults or in other countries.

A smaller number of articles suggested factors derived from molecular weights (equimolar). Obtaining such factors is straightforward using publicly available mole weights. These factors were basically independent from the other approaches. Results in terms of dose effects in late effects research using these factors may not be comparable to results based on data using effect equivalence‐based factors.

These conversion factors in general and their underlying principles potentially have great value for research with aggregated data, such as epidemiological late effects research.

## AUTHOR CONTRIBUTIONS

**Meike Ressing:** Conceptualization (equal); data curation (equal); formal analysis (equal); investigation (equal); methodology (equal); software (equal); validation (equal); visualization (equal); writing – original draft (lead); writing – review and editing (lead). **Cornelia Becker:** Conceptualization (equal); investigation (equal); methodology (equal); project administration (equal); writing – review and editing (supporting). **Christian Müller:** Investigation (equal); resources (equal); validation (equal); writing – review and editing (supporting). **Seyed Hamidreza Mahmoudpour:** Methodology (equal); validation (equal); writing – review and editing (supporting). **Gabriele Calaminus:** Investigation (equal); resources (equal); writing – review and editing (supporting). **Thorsten Langer:** Investigation (equal); resources (equal); writing – review and editing (supporting). **Friederike Erdmann:** Funding acquisition (equal); supervision (equal); writing – review and editing (supporting). **Mathias Voigt:** Data curation (equal); writing – review and editing (supporting). **Melanie Kaiser:** Data curation (equal); writing – review and editing (supporting). **Peter Kaatsch:** Conceptualization (equal); funding acquisition (equal); resources (equal); supervision (equal); writing – review and editing (supporting). **Maria Blettner:** Conceptualization (equal); funding acquisition (equal); resources (equal); supervision (equal); writing – review and editing (supporting). **Claudia Spix:** Conceptualization (equal); formal analysis (equal); funding acquisition (equal); investigation (equal); methodology (equal); project administration (equal); resources (equal); supervision (equal); visualization (equal); writing – original draft (supporting); writing – review and editing (supporting).

## FUNDING INFORMATION

This study was funded by Deutsche Krebshilfe, Grant Number: 70112099.

## CONFLICT OF INTEREST STATEMENT

Seyed Hamidreza Mahmoudpour is currently an employee of Merck KGaA, Darmstadt, Germany. All other authors have stated explicitly that there are no conflicts of interest in connection with this article.

## ETHICS STATEMENT

The authors declare that this work has been done in accordance to Wiley “Best Practice Guidelines on Research Integrity and Publishing Ethics” and that is has been performed in an ethical and responsible way, with no research misconduct, which includes, but is not limited to data fabrication and falsification, plagiarism, image manipulation, unethical research, biased reporting, authorship abuse, redundant or duplicate publication, and undeclared conflicts of interest

## Supporting information


**Data S1:** Supporting Information.

## Data Availability

Data available on request from the authors.
