# Supplementary material for: Equivalent doses for anticancer agents used in pediatric oncology: A literature review and evaluation of a novel approach for conversion factors
Source: Cancer Rep (Hoboken). 2023 Mar 28;6(5):e1811. doi: 10.1002/cnr2.1811 (PMC10172172; doi:10.1002/cnr2.1811)
Supplement: Supplementary file 1 — Data S1: Supporting Information. [file CNR2-6-e1811-s001.docx]

**Supplementary Table 1 MEDLINE search query**

| **Search** | **Keywords used** | **Resulting query** |
| --- | --- | --- |
| **Search 1** | **childhood second cancer AND chemotherapy AND dose** | ("childhood"[All Fields] OR "childhoods"[All Fields]) AND ("neoplasms, second primary"[MeSH Terms] OR ("neoplasms"[All Fields] AND "second"[All Fields] AND "primary"[All Fields]) OR "second primary neoplasms"[All Fields] OR ("second"[All Fields] AND "cancer"[All Fields]) OR "second cancer"[All Fields]) AND ("chemotherapy s"[All Fields] OR "drug therapy"[MeSH Terms] OR ("drug"[All Fields] AND "therapy"[All Fields]) OR "drug therapy"[All Fields] OR "chemotherapies"[All Fields] OR "drug therapy"[MeSH Subheading] OR "chemotherapy"[All Fields]) AND "dose"[All Fields] |
| **Search 2** | **cortisone AND equivalence dose** | ("cortisone"[MeSH Terms] OR "cortisone"[All Fields] OR "cortisones"[All Fields]) AND (("equivalence"[All Fields] OR "equivalences"[All Fields] OR "equivalencies"[All Fields] OR "equivalency"[All Fields] OR "equivalent"[All Fields] OR "equivalently"[All Fields] OR "equivalents"[All Fields]) AND "dose"[All Fields]) |

**Supplementary Figure 1 Scatter Plot and Regression Line of the Factor based on Effect Equivalence from Literature Review and the Factor based on Typical dose (excluding three substances strongly influencing the slope*)**


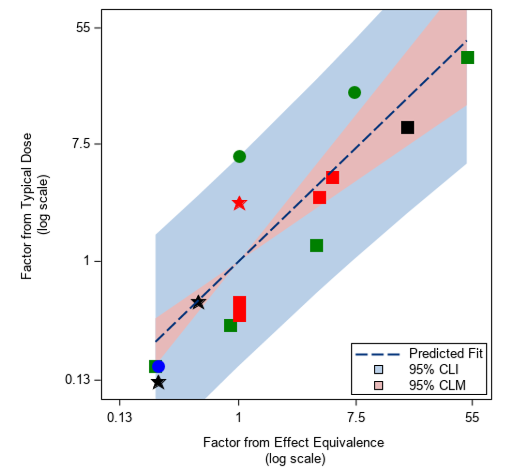


**
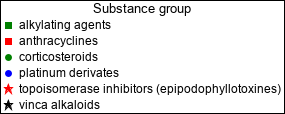
**

Correlation coefficient r = 0.89

CLI: confidence limits for the individual predicted values

CLM: confidence limits for the mean predicted values

* Melphalan (ATC-code L01AA03), Busulfan (L01AB01), and Lomustine (L01AD02)

**Supplementary Figure 2 Scatter Plot and Regression Line of the Factor based on Effect Equivalence from Literature Review and the Factor based on Typical dose, excluding Glucocorticoids**


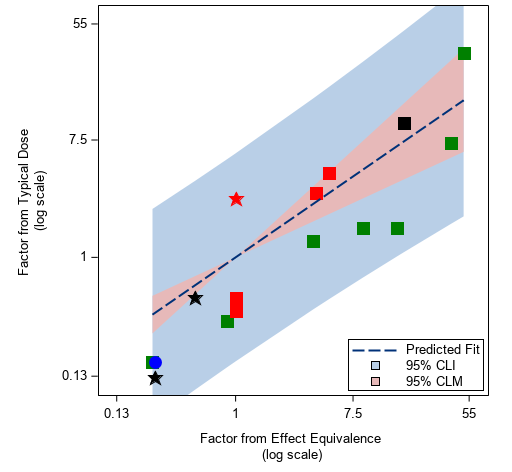


**
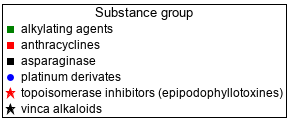
**

Correlation coefficient r = 0.91

CLI: confidence limits for the individual predicted values

CLM: confidence limits for the mean predicted values

**Supplementary Figure 3 Scatter Plot and Regression Line of the Factor based on Effect Equivalence from Literature Review and the Factor derived from the Molecular Weights of the Substances**


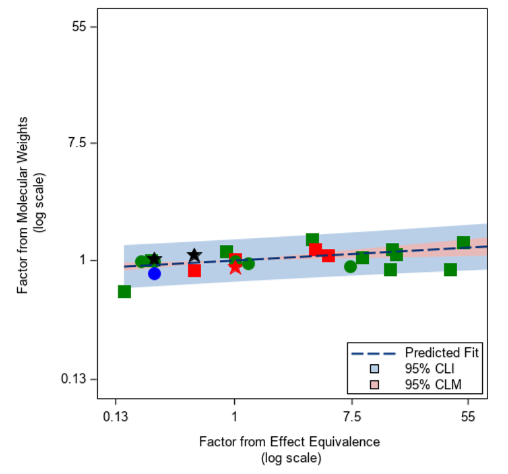


**
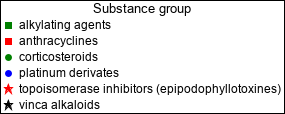
**

Correlation coefficient r = 0.54

CLI: confidence limits for the individual predicted values

CLM: confidence limits for the mean predicted values

**Supplementary Figure 4 Scatter Plot and Regression line of Factor from Typical Dose* and the Factors derived from the Molecular Weights of the Substances**


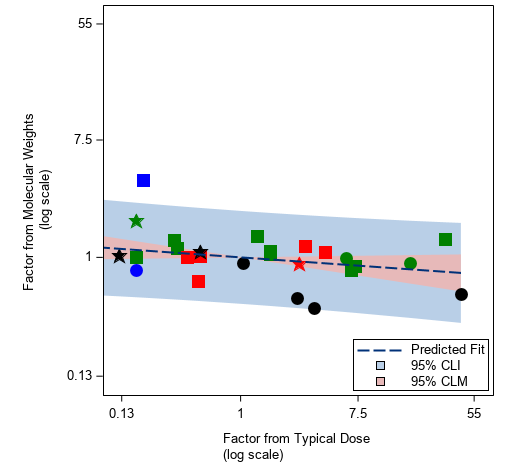


**
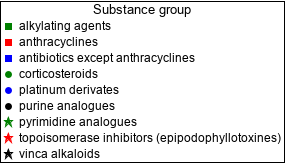
**

Correlation coefficient r = -0.32

CLI: confidence limits for the individual predicted values

CLM: confidence limits for the mean predicted values

* factors derived from treatment protocols in pediatric oncology in Germany
